# Expression and potential regulatory mechanism of cellular senescence-related genes in Alzheimer’s disease based on single-cell and bulk RNA datasets

**DOI:** 10.3389/fnins.2025.1595847

**Published:** 2025-05-21

**Authors:** Dujuan Sha, Jingxuan Zhang, Xu Fang, Xinyu Wang, Xuan He, Xin Shu

**Affiliations:** ^1^Department of General Practice, Nanjing Drum Tower Hospital Clinical College of Nanjing University of Chinese Medicine, Nanjing, China; ^2^Department of General Practice, Nanjing Drum Tower Hospital, Affiliated Hospital of Medical School, Nanjing University, Nanjing, China; ^3^The State Key Laboratory of Pharmaceutical Biotechnology, Nanjing University, Nanjing, China

**Keywords:** Alzheimer’s disease, cellular senescence, gene, single-cell RNA, bulk RNA datasets

## Abstract

**Introduction:**

Alzheimer’s disease (AD) is the most common cause of dementia in the elderly. However, the particular cause of AD development has not been fully elucidated. Currently, cellular senescence is recognized as a contributing factor to the aging process and age-related diseases.

**Methods:**

The present study aimed to identify the hinge of regulatory factors in dysfunctional cellular senescence genes in AD via integrating multiple omics analysis, including single-cell RNA sequencing and bulk sequencing data. In addition, UMAP scatter diagrams were constructed, while active cell subtypes and pathways involved in cellular senescence were identified via performing Gene Ontology (GO) and Kyoto Encyclopedia of Genes and Genomes (KEGG) pathway enrichment analysis, respectively.

**Results:**

The results indicated that a total of seven clusters were detected by known marker genes, including excitatory neurons, inhibitory neurons, astrocytes, microglial cells, oligodendrocytes, oligodendrocyte progenitor cells and pericytes/endothelial cells. *CDK18* was specifically expressed in oligodendrocytes, *RUNX1* in microglia, *SORBS2* and *KSR2* in neurons, *PDZD2* in oligodendrocyte progenitors, *YAP1* in astrocytes and *NOTCH3* in pericytes/endothelial cells. Astrocytes, microglia, and pericytes/endothelial cells were found to be the most active cell subtypes. AD-associated cellular senescence genes in the Astrocytes subgroup (*SOX5*, *AR*, *HMGB1*, *NR2E1*, *ID4*, *TP53*, *MXD4*, *FOS*, *BHLHE40*, *PIVEP1*), microglia subgroup (*BCL6*, *ETS2*, *CEBPB*, *MXD4*, *FOS*, *NFE2L2*, *FOXO3*, *IRF3*, *PBRM1*, *RUNX1*, *IRF5*, *ZNF148*) and pericyte/endothelial cell subgroup (*SOX5*, *BCL6*, *ETS2*, *CEBPB*, *FOS*, *TP63*, *TBX2*, *ETS1*, *BHLHE40*, *ID1*) were identified. Furthermore, potential therapeutic targets and drugs for AD were identified via analyzing the molecular mechanisms and pathways involved in cellular senescence.

**Conclusion:**

The above findings demonstrated that cellular senescence could play a crucial role in the pathogenesis of AD and highlighted the significance of understanding the role of cellular senescence in the pathogenesis of AD. The results of the current study could provide novel insights into the development of potential therapeutic targets and pave the way for the development of novel therapeutic strategies for AD.

## 1 Introduction

Alzheimer’s disease (AD) is a debilitating neurodegenerative disorder that affects the elderly and elderly with pre-dementia, thus affecting millions of people worldwide (Scheltens et al., 2021; Lopez-Lee et al., 2024). This disease is characterized by progressive cognitive decline, memory loss, language difficulties and changes in behavior and personality (Rajendran and Krishnan, 2024). AD is the most common cause of dementia in the elderly, accounting for 60–70% of all dementia cases. Pathologically, the disease is characterized by the accumulation of amyloid-*β* (Aβ) plaques and neurofibrillary tangles (NFTs), composed of hyperphosphorylated tau protein, in the brain (Ma et al., 2022; Ossenkoppele et al., 2022). These pathologies are accompanied by chronic inflammation, oxidative stress and synaptic dysfunction, thus leading to synaptic loss and neuronal death (Rostagno, 2022; Jucker and Walker, 2023; Twarowski and Herbet, 2023). While the particular cause of AD has not been fully understood, it is widely accepted that genetic and environmental factors play a key role in its development (Ogbodo et al., 2022).

Cellular senescence is a complex process that results in irreversible cell cycle arrest and the acquisition of a distinct phenotype in response to various stimuli, including DNA damage, oxidative stress and oncogene activation (Sharma et al., 2020). This phenomenon was first described over half a century ago. Currently, significant advances have been made in the understanding of the molecular mechanisms and pathways involved in the development and maintenance of cellular senescence (Roger et al., 2021; Liu, 2022). There are several types of cellular senescence (Mohamad Kamal et al., 2020), including replicative senescence, stress-induced premature senescence (SIPS), oncogene-induced senescence (OIS), therapy-induced senescence (TIS), immunosenescence and senescence associated with chronic inflammation.

The phenotype of senescent cells is characterized by an enlarged and flattened morphology, increased senescence-associated *β*-galactosidase (SA-β-Gal) activity and the activation of several genes and pathways, including those of the p53 and p16INK4a tumor suppressor pathways. Additionally, senescent cells display a senescence-associated secretory phenotype (SASP) and are resistant to apoptosis, while they are also characterized by the activation of metabolic and endoplasmic reticulum stress-related pathways. While it was initially believed that senescence could be a protective mechanism against cancer development, it is now recognized as a contributing factor to the aging process and age-related diseases (Birch and Gil, 2020; Guerrero et al., 2021; Zhang et al., 2022; Holloway et al., 2023).

Recent studies suggest that cellular senescence plays a key role in the pathological development and progression of AD (Holloway et al., 2023). Several senescence-associated features are detected in the brains of AD patients. The relationship between cellular senescence genes and AD is garnering increasing attention. Advances in the biological analysis of cellular senescence in aging and disease could provide a comprehensive understanding of the pathways involved in this particular process (Avelar et al., 2020). Therefore, identifying differentially expressed genes (DEGs) and signaling pathways could provide valuable insights into the molecular mechanisms underlying cellular senescence.

The present study aimed to explore the dysfunctional mechanism of cellular senescence in AD by joint analysis of single-cell RNA (scRNA) and bulk sequencing data to identify the key transcription factors, thus assisting the development of potential therapeutic targets and drugs for AD.

## 2 Materials and methods

### 2.1 Sequencing data processing

A portion of 10x genomic scRNA sequencing data from the frontal cortex tissues and bulk sequencing data from the frontal cortex tissues were downloaded from the Gene Expression Omnibus database (GSE174367). A total of 11 AD samples and seven control samples were included in the scRNA sequencing data, while the bulk sequencing data composed of 47 AD samples and 48 control samples (Supplementary Table S1; Supplementary Figure S1). Cells with <200 or >10,000 expressed genes and a mitochondrial gene proportion of >10% were filtered out. Principal component analysis (PCA) was performed using the R package ‘Seurat’ (version 4.1.1). The data were normalized by the normalize Data function and the top 2,000 highly variable genes were identified and scaled by the Find Variable Features and Scale Data functions, respectively. The Principal components (PCs) were identified by the RUNPCA function and the top 20 PCs were selected by the Elbow Plot function. Package ‘harmony’ (version 0.1.1) in R was used to remove the batch effects from the single-cell gene expression data, which could disrupt the downstream analysis (Korsunsky et al., 2019). Clustering analysis was performed via applying the Find Neighbors and Find Clusters functions, followed by the visualization of the clustering results using the Run UMAP function (Zhao et al., 2022). Finally, the Find All Markers function was used to identify highly expressed genes specific to different cell subtypes, followed by cell subtype annotation using classical markers. The differential expression analysis of the bulk sequencing data was carried out using the rank-based test, with a filtering threshold of *p* < 0.05 and |log2 Fold Change (FC)| > 0.2. To identify DEGs between each cell subtype, the Find All Markers function was used for the scRNA seq data (Sinha et al., 2018). A min.pct of 0.1 and logfc. Threshold of 0.25 were set, while only genes with *p* < 0.05 and only. Pos = TRUE were retained. Multiple imputation was used to handle missing data.

### 2.2 Evaluating the activity score of cellular senescence-related genes by AUCell

CellAge data (https://genomics.senescence.info/cells/) is used to analyze the expression of aging-related genes and evaluate the activity score of each gene in individual cells based on single-cell RNA sequencing data. The activity score is used to define the expression patterns and trends of aging-related genes in different types of cells. CellAge data was rigorously constructed following a scientific literature search, manual curation, and annotation process. The inclusion of genes in the database was based on criteria similar to those outlined by Avelar et al. (2020). The CellAge database contains 279 known human genes associated with cellular senescence. The Add Module Score function in the ‘Seurat’ R package was used to calculate the scores of the cell aging-related genes in each cell subtype. The active cell subtypes were determined based on the score distribution density curve and their distribution in the UMAP plots.

### 2.3 Functional enrichment analysis

The functional annotations and pathways of the cellular senescence-related genes were imported from Gene Ontology (GO) and Kyoto Encyclopedia of Genes and Genomes (KEGG) databases (Kanehisa and Goto, 2000; Kanehisa, 2019). Package ‘Cluster Profile’ (Wu et al., 2021) in R was used to perform functional enrichment analysis. Multiple hypothesis testing on the *p values* was carried out based on Benjamini-Hochberg False Discovery Rate (FDR).

### 2.4 Expression profile of common genes between cellular senescence and AD

The bulk sequencing data revealed that both the active cell subpopulation-specific highly expressed genes and DEGs were significantly enriched in particular functions, such as immune response. Therefore, the current study focused on studying the intersection of these genes with cell senescence-related genes to obtain the AD-related cellular senescence genes. The Package ‘ggvenn’ in R was used to visualize the intersection of bulk RNA-seq DEGs, active cell subpopulation-specific highly expressed genes and cell senescence-related genes. To investigate the transcriptional regulation of the genes in the interactions, the corresponding transcription factors were obtained from the HumanTFDB database (http://bioinfo.life.hust.edu.cn/HumanTFDB) and visualized by Venn diagram.

### 2.5 Assessment of the regulatory effect of non-coding RNAs

To explore the regulatory effect of non-coding RNAs on the expression of common cell senescence-related genes, the micro (mi)RNA-mRNA regulatory targeting associations were obtained by the StarBase database (Li et al., 2014). Therefore, a non-coding RNA regulatory network for AD-associated cellular senescence genes was constructed.

### 2.6 Potential therapeutic drugs

Based on the AD-associated cellular senescence genes, the Drug-Gene Interaction database (DGIdb) (Freshour et al., 2021) (https://www.dgidb.org/) was used to identify potential therapeutic drugs via gene-drug interactions. Filter drug-gene interactions for high confidence by selecting those with a score greater than 3(Score>3).The drug-gene interactions were visualized by Sankey diagrams, which were plotted using the ‘ggalluvial’ and ‘reshape’ packages in R.

## 3 Results

### 3.1 Heterogeneity in AD by single-cell transcriptomics

**Figure 1:**
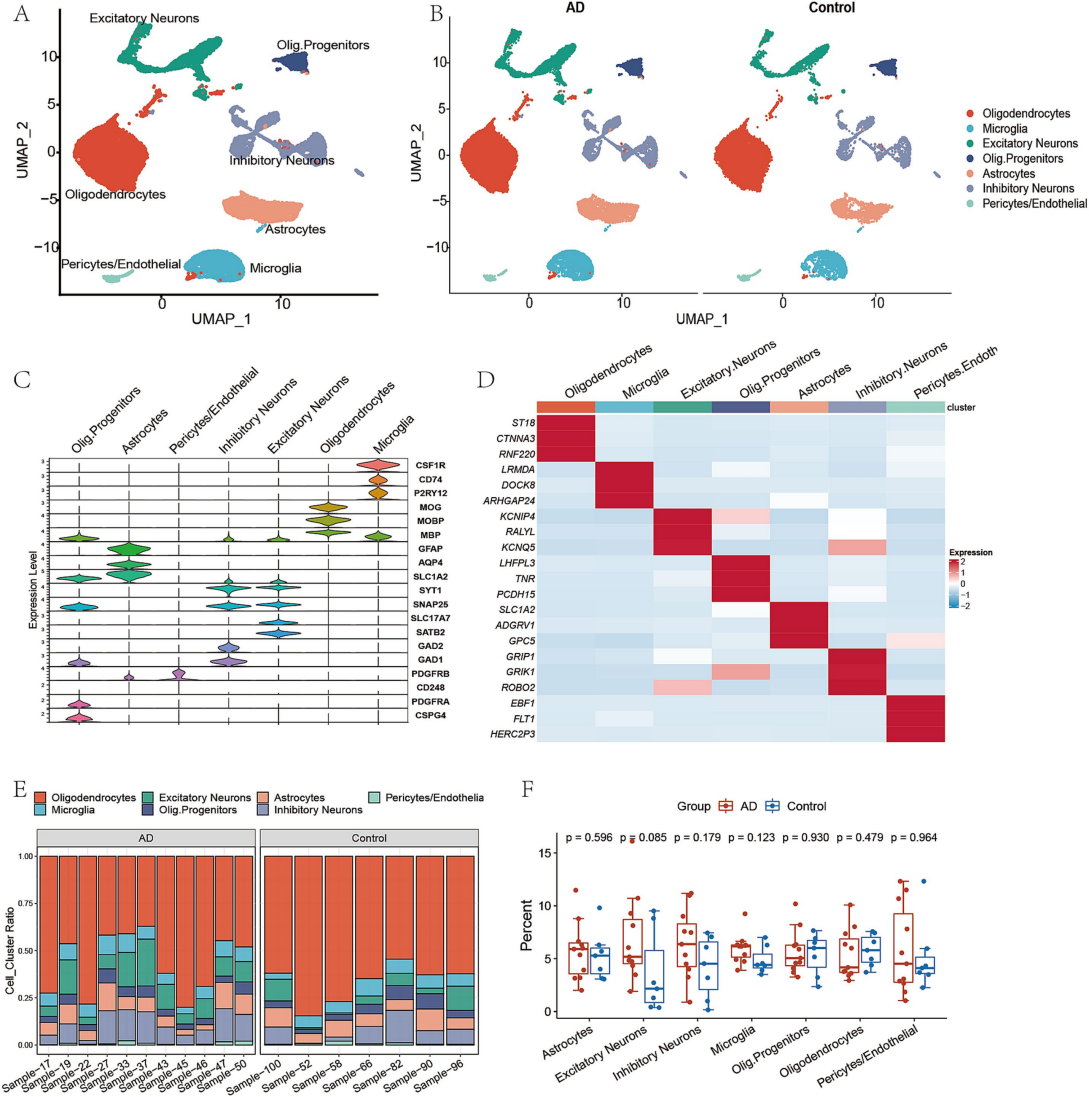
Heterogeneity in Alzheimer’s disease by single-cell transcriptomics. **(A)** UMAP scatter diagram of scRNA sequencing gene expression. Different colors represent different cell types. **(B)** UMAP scatter diagram of different groups. **(C)** The expression distribution curve of marker genes in violin plot. **(D)** Heatmap of top 3 subtypes of DEGs. **(E)** Compositions of cell types in each sample. **(F)** Boxplot of the proportion changes for each cell type.

The scRNA sequencing data of the prefrontal cortex tissues from the GSE174367 dataset, consisted of 11 late-stage AD samples and seven normal control samples. Sequencing data were analyzed from a total of 61,472 cells, including 38,676 and 22,796 cells from AD and control samples, respectively. The filtered single-cell data were subjected to dimension reduction and clustered into seven subtypes annotated by known marker genes (Figure 1A). The above subtypes included 5,990 excitatory neurons (*SNAP25*, *SYT1*, *SLC17A7*, *SATB2*), 5,874 inhibitory neurons (*SNAP15*, *SYT1*, *GADl*, *GAD2*), 4,794 astrocytes (*GFAP*, *AQP4*, *SLC1A2*), 4,198 microglial cells (*CSFlR*, *CD74*, *P2RY12*), 37,398 oligodendrocytes (*MOBP*, *MBP*, *MOG*), 2,731 oligodendrocyte progenitor cells (*PDGFRA*, *CSPG4*) and 487 pericytes/endothelial cells (*PDGFRB*, *CD248*). The identified seven cell subtypes were visualized by a UMAP scatter plot (Figures 1A,B). The marker genes for each subtype are presented in violin plots (Figure 1C). Additionally, the heatmap of the high expressed genes in each subtype is shown in Figure 1D. The composition and proportion of each cell subtype in the AD and control samples were calculated and compared (Figures 1E,F). The most abundant cell subtype in all samples was oligodendrocytes. Although there was a difference in the proportions of the seven cell subtypes between the AD and control samples, statistical significance was not reached.

### 3.2 Dysregulation of cellular senescence via single-cell transcriptomic analysis

**Figure 2:**
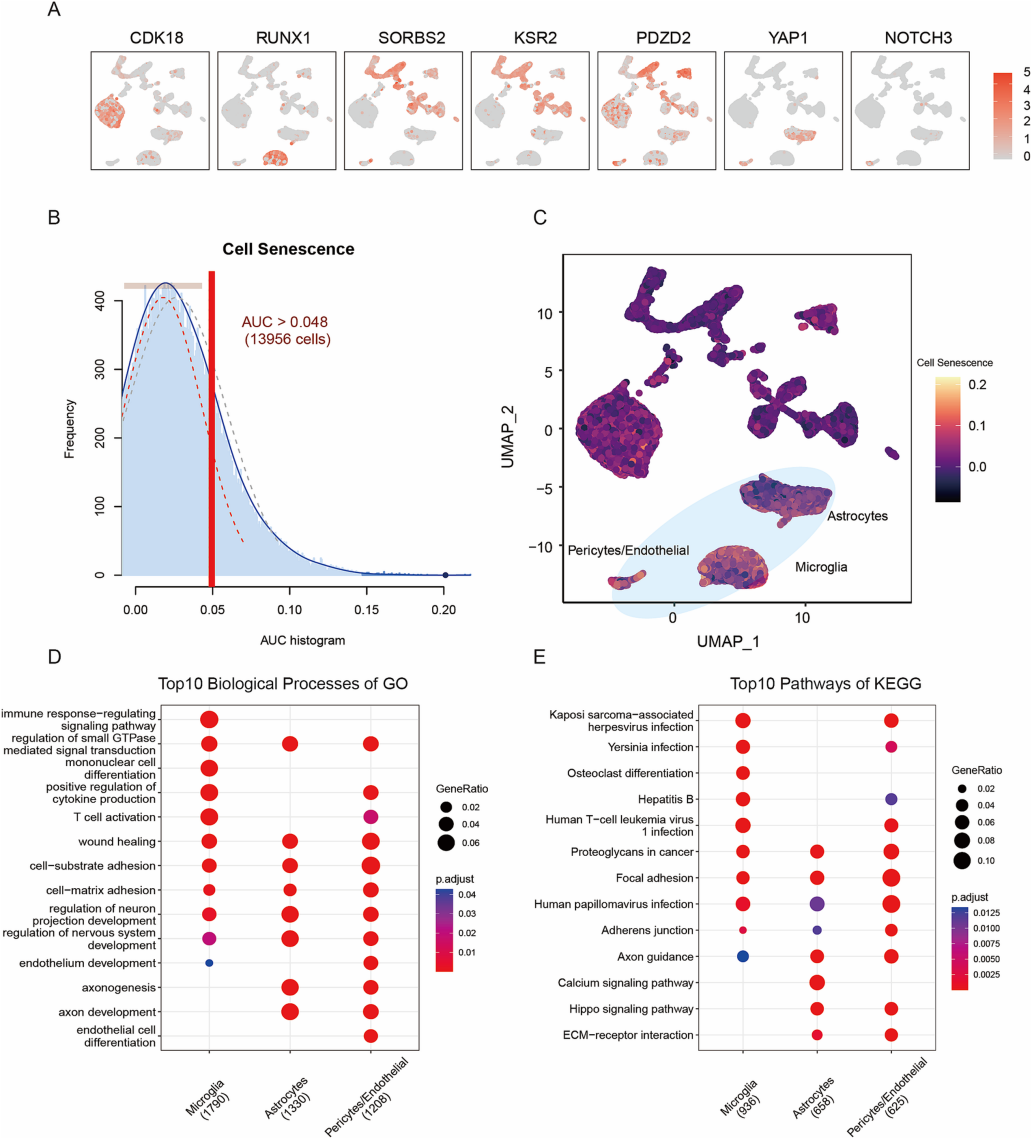
Dysregulation of cellular senescence by transcriptomic analysis. **(A)** UMAP scatter diagrams of cellular senescence gene expression. **(B)** Activity score distribution of cellular senescence genes. **(C)** UMAP scatter diagrams of activity scores of cellular senescence genes. **(D)** GO BP analysis results of active cell subtypes. **(E)** KEGG pathway results of active cell subtypes.

To investigate the expression characteristics of the cellular senescence-related genes at the single-cell level, a total of 279 senescence-related genes were obtained from the Cell Age database. These senescence-related genes were specifically expressed in particular cell types (Figure 2A). For example, *CDK18* was specifically expressed in oligodendrocytes, *RUNX1* in microglia, *SORBS2* and *KSR2* in neurons, *PDZD2* in oligodendrocyte progenitors, *YAP1* in astrocytes and *NOTCH3* in pericytes/endothelial cells. Furthermore, to determine the active cell population the Add Module Score function in the ‘Seurat’ R package was used to calculate the activity score, with a threshold of 0.048, of the senescence-related genes in each cell subtype. The threshold was calculated using the AUCell algorithm (Zhao et al., 2023). The results showed that cells with enhanced expression of senescence-related genes displayed higher activity scores. Therefore a total of 13,956 cells showed relatively high gene set activity scores. Among the cell subtypes examined, Astrocytes, microglia, and pericytes/endothelial cells were identified as the most active cell subtypes (Figures 2B,C). Furthermore, to determine the functions of the active cell populations, GO and KEGG pathway functional enrichment analysis was performed on the cell subtype-specific highly expressed genes, sorted by the p.adjust function (Figures 2D,E). The annotation results from the GO and KEGG pathway enrichment analysis are listed in Supplementary Table S2. Microglia was mainly enriched in the terms ‘immune response-regulating signaling pathway’, ‘regulation of small GTPase mediated signal transduction’, ‘mononuclear cell differentiation’, ‘positive regulation of cytokine production’, ‘T cell activation’ and ‘positive regulation of cell adhesion’. Astrocytes were mainly involved in the biological processes (BPs) of ‘axonogenesis’, ‘axon development’, ‘regulation of nervous system development’, ‘regulation of neuron projection development’, ‘axon guidance’, ‘neuron projection guidance’, ‘regulation of neurogenesis’ and ‘neural precursor cell proliferation’. Finally, pericytes/endothelial cells were mainly annotated in ‘cell-substrate adhesion’, ‘endothelium development’, ‘endothelial cell differentiation’, ‘wound healing’ and ‘cell-matrix adhesion’. The results of the functional differential analysis of the active cell subtypes between AD and control samples are shown in Supplementary Figure S2.

### 3.3 Expression characteristics of AD-related genes based on bulk sequencing data

**Figure 3:**
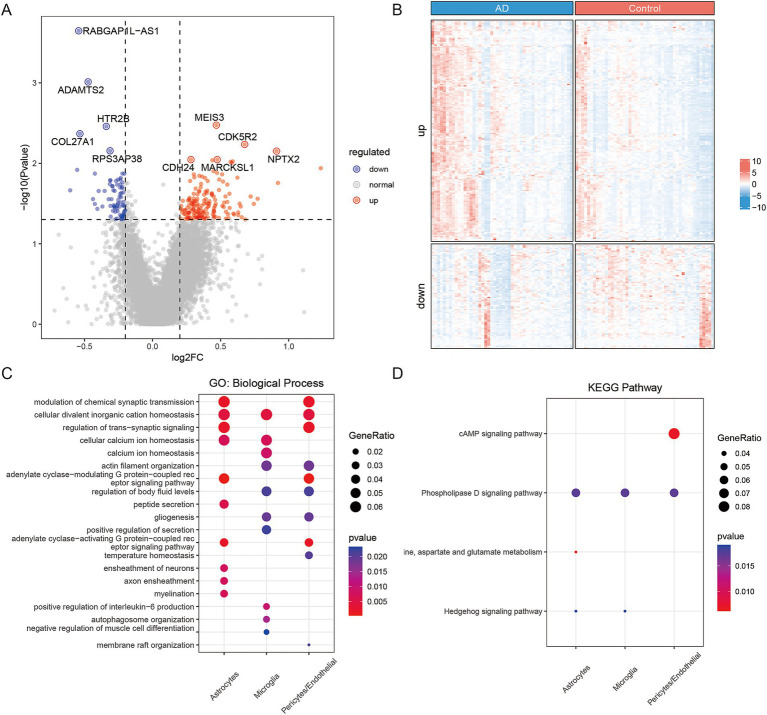
Gene expression characteristics of AD based on bulk sequencing data. **(A)** Volcano plot of DEGs (*n* = 240). Red dots are the upregulated DEGs, and blue dots are downregulated DEGs. **(B)** Heatmap of expression level of DEG’s. **(C)** Top 10 subtypes of the intersection between GO BP results of DEGs and active subtype results of single cells. **(D)** The intersection between KEGG pathway results of DEGs and active subtype results of single cells.

A total of 240 significantly DEGs were identified from the bulk sequencing data (Figures 3A,B; Supplementary Table S3). Compared with the control normal reference group, 163 upregulated and 77 downregulated genes were detected in the AD samples. The functional enrichment analysis of DEGs showed similar results as those obtained in the scRNA sequencing analysis of the active cell subtypes (Figures 3C,D). Therefore, the common functions in the GO BP results were ‘adenylate cyclase-modulating G protein-coupled receptor signaling pathway’, ‘modulation of chemical synaptic transmission’, ‘regulation of trans-synaptic signaling’, ‘cellular divalent inorganic cation homeostasis’, ‘myelination’, ‘cellular calcium ion homeostasis’, ‘ensheathment of neurons’, ‘actin filament organization’, ‘gliogenesis’ and ‘regulation of cytosolic calcium ion concentration’. In addition, the KEGG pathway enrichment analysis results revealed that the AD-related genes were mainly enriched in the terms ‘alanine, aspartate and glutamate metabolism’, ‘cAMP signaling pathway’, ‘phospholipase D signaling pathway’ and ‘hedgehog signaling pathway’. The full results according to the *p* values are shown in Supplementary Table S4.

### 3.4 Expression characteristics and regulatory networks of AD-related cellular senescence genes

**Figure 4:**
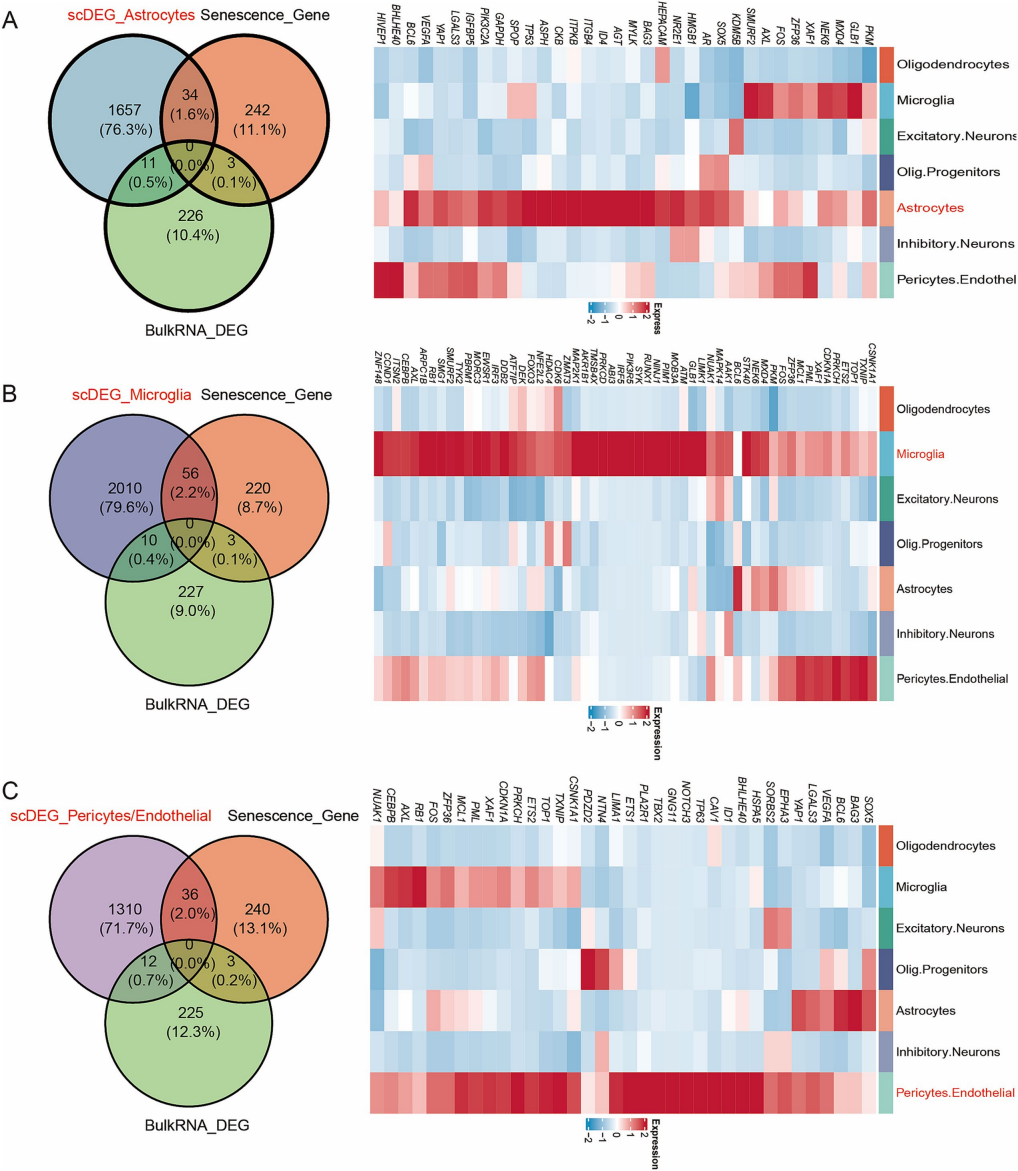
Expression characteristics and of AD-associated cellular senescence genes. **(A)** Venn diagram of scRNA sequencing DEGs, bulk sequencing DEGs and cellular senescence genes in astrocytes subtype. **(B)** Venn diagram of scRNA sequencing DEGs, bulk sequencing DEGs and cellular senescence genes in microglia subtype. **(C)** Venn diagram of scRNA sequencing DEGs, bulk sequencing DEGs and cellular senescence genes in pericytes/endothelial subtype.

**Figure 5:**
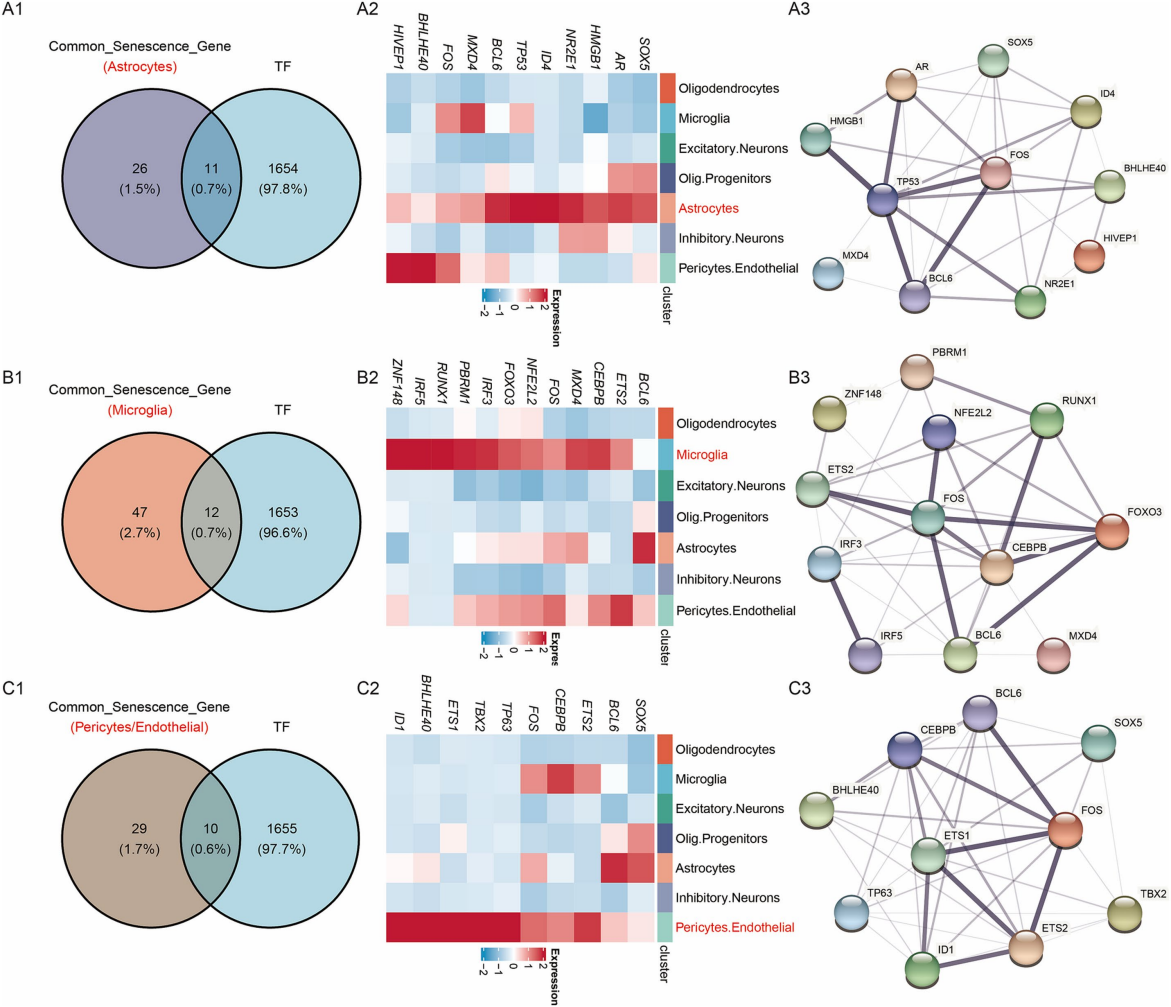
Regulatory networks of AD-associated cellular senescence genes. **(A1)** Interaction between AD-associated cellular senescence genes and HumanTFs in astrocytes. **(A2)** Heatmap of the gene expression of the interaction in scRNA sequencing data. **(A3)** PPI network diagram. **(B1)** Interaction between AD-associated cellular senescence genes and HumanTFs in microglia. **(B2)** Heatmap of the gene expression of the interaction in scRNA sequencing data. **(B3)** PPI network diagram. **(C1)** Interaction between AD-associated cellular senescence genes and HumanTFs in pericytes/endothelial. **(C2)** Heatmap of the gene expression of the interaction in scRNA sequencing data. **(C3)** PPI network diagram.

**Figure 6:**
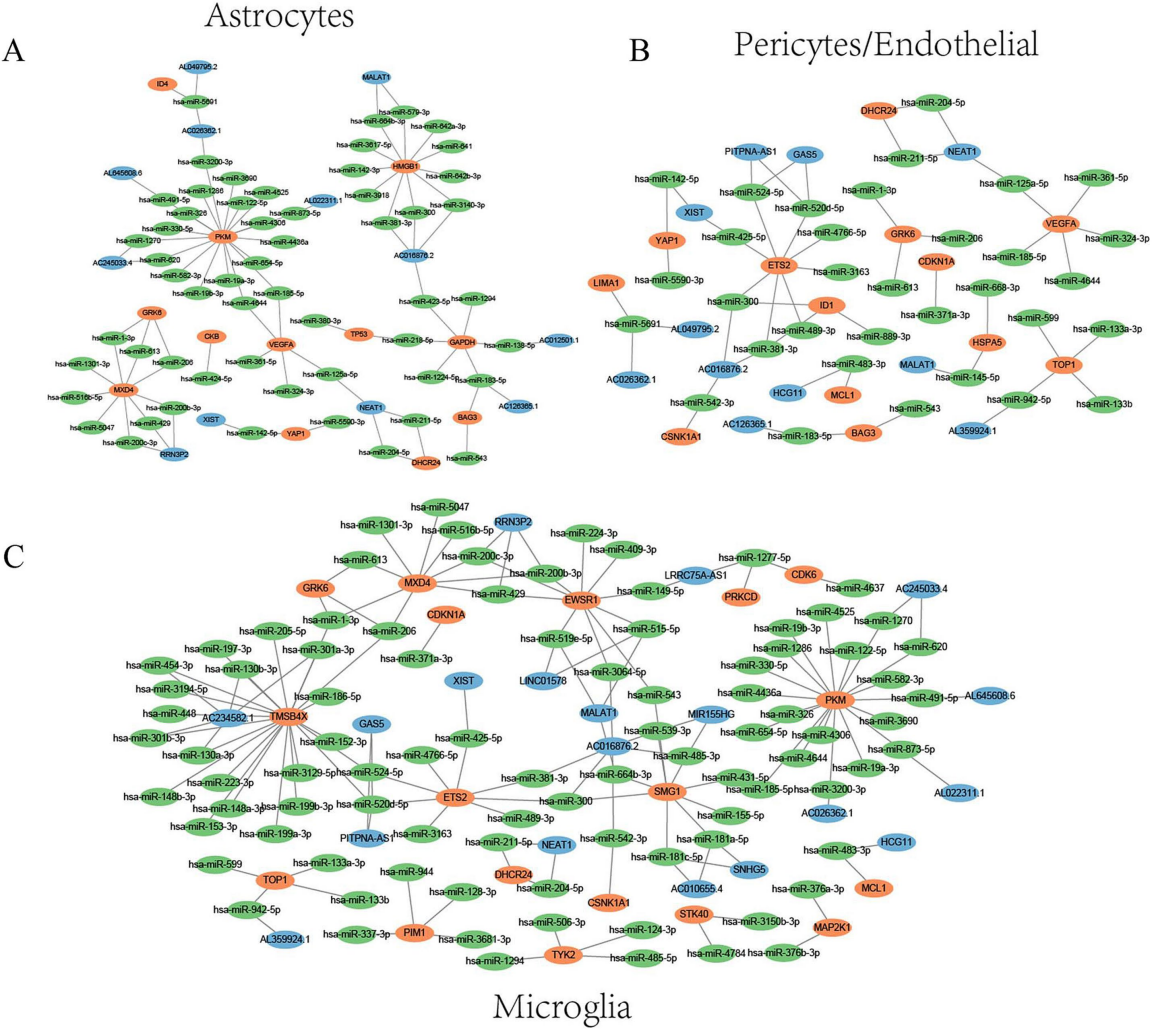
Regulatory networks of lncRNA (Blue), miRNA (Green) and AD-associated cellular senescence genes (Red). **(A)** Astrocytes. **(B)** Pericytes/Endothelial. **(C)** Microglia.

As shown in Figure 4, genes in the active cell subtypes and DEGs in the bulk sequencing data shared common functional enrichment annotations. The current study focused on the active cell subtype-specific gene set, bulk sequencing DEGs and cellular senescence genes. However, since there was no overlapping among these three gene sets (Figure 4A), the AD-associated cellular senescence genes were defined by the interaction between the cellular senescence genes and active cell subtype-specific genes or bulk sequencing DEGs. A total of 37 AD-related cellular senescence genes were identified in the astrocyte subtype, including 34 subtype-specific genes and three bulk sequencing DEGs (Figure 4A). Additionally, a total of 59 AD-associated cellular senescence genes were detected in the microglia subtype, including 56 subtype-specific genes and three bulk sequencing DEGs (Figure 4B). The 39 AD-associated cellular senescence genes in pericytes/endothelial cells, encompassed 36 subtype-specific genes and three bulk sequencing DEGs (Figure 4C). To investigate the transcriptional regulation of AD-associated cellular senescence genes, 1,665 human transcription factors were downloaded from the HumanTFDB database. Therefore, 11, 12 and 10 AD-associated cellular senescence genes were detected in astrocyte (*SOX5*, *AR*, *HMGB1*, *NR2E1*, *ID4*, *TP53*, *MXD4*, *FOS*, *BHLHE40*, *PIVEP1*) (Figures 5A1,A2), microglia (*BCL6*, *ETS2*, *CEBPB*, *MXD4*, *FOS*, *NFE2L2*, *FOXO3*, *IRF3*, *PBRM1*, *RUNX1*, *IRF5*, *ZNF148*) (Figures 5B1,B2) and pericyte/endothelial cell (*SOX5*, *BCL6*, *ETS2*, *CEBPB*, *FOS*, *TP63*, *TBX2*, *ETS1*, *BHLHE40*, *ID1*) (Figures 5C1,C2) subgroups, respectively. Furthermore, to construct a protein–protein interaction (PPI) network among transcription factors, the STRING database (version 11.5; https://cn.string-db.org) was utilized (Figures 5A3,B3,C3). The minimum confidence threshold score was 0.4. Finally, to investigate how non-coding RNAs could regulate AD-associated cellular senescence genes, a regulatory network including the long non-coding (lnc) RNA-miRNA interactions, was constructed (Figure 6). 12, 19 and 11 AD-associated cellular senescence lncRNA were detected in astrocyte (*AL049795.2, AC026362.1, AL645608.6, AC245033.4, AL022311.1, RRN3P2, XIST, MALAT1, AC016876.2, NEAT1, AC126365.1, AC012501.1*) (Figure 6A), microglia (*AC234582.1, AL359924.1, GAS5, PITPNA-AS1, XIST, RRN3P2, LINC01578, NEAT1, MALAT1, LRRC75A, AC016876, MIR155HG, AC010655.4, SNHG5, AC026362.1, HCG11, AC245033.4, AL022311.1, AL645608.6*) (Figure 6B) and pericyte/endothelial cell (*AC026362.1, AL049795.2, XIST, AC016876.2, AC126365.1, HCG11, PITPNA-AS1, GAS5, MALAT1, NEAT1, AL359924.1*) (Figure 6C) subgroups, respectively. 4 lncRNA (AC026362.1, XIST, MALAT1, NEAT1) potentially regulate cellular senescence in all three cell subtypes via different mechanisms. This network was created using data from the publicly available StarBase database (Li et al., 2014).

### 3.5 Potential targeted therapies

Based on the AD-associated cellular senescence genes, the Gene-Drug interaction data in the DGIdb database were used to identify potential therapeutic drugs. The high-confidence interactions were filtered using a drug-gene interaction score of >3. 9 drugs for astrocytes, 15 for microglia, and 11 for pericytes/endothelial cells were identified. High confidence for astrocytes (*GAPDH*, *GRK6*, *HMGB1*, VEGFA), microglia (*CDK1*, *CDKN1A*, *CSNK1A1*, *EWSR1*, *GRK6*, *PIM1*, *PRKCD*, *TOP1*), and pericytes/endothelial cells (*CDKN1A*, *CSNK1A1*, *GRK6*, *HSPA5*, *TOP1*, *VEGFA*) were identified. The interactions between drugs and genes were visualized using a Sankey diagram. The full results are shown in Supplementary Figure S3 and Supplementary Table S5.

## 4 Discussion

Cellular senescence is a process by which cells permanently exit the cell cycle and cease dividing. It is believed that this process plays a crucial role in both aging and the development of age-related diseases. The accumulation of senescent cells over time can contribute to the decline in tissue function and the development of age-related pathologies. Recent research has focused on identifying specific signaling pathways involved in cellular senescence, as well as potential interventions aiming at reducing the burden of senescent cells in the aging population (Herdy et al., 2022; Sharma et al., 2022; Awad et al., 2023).

It has been reported that cellular senescence is involved in the pathogenesis of AD (Guerrero et al., 2021; Singh and Bhatt, 2024). Therefore, previous studies reported the accumulation of senescent cells in the brains of patients with AD, accompanied by p16INK4a and p53 upregulation, two senescence-associated markers. Senescent cells are also involved in neuroinflammation, oxidative stress and accumulation of amyloid-beta protein in patients with AD. Furthermore, targeting cellular senescence could provide a novel therapeutic approach for AD, since it has been reported that removing senescent cells can reduce the burden of amyloid-beta and improve cognitive function in mouse models of AD.

Dysregulation of cellular senescence-related genes has been implicated in various age-related diseases, including cancer, cardiovascular and neurodegenerative diseases. The present study identified that dysregulation of cellular senescence genes could be a crucial factor involved in the development and maintenance of AD, thus providing a foundation for future research aimed at understanding the role of cellular senescence in other age-related diseases.

Herein, key genes and signaling pathways were identified in AD by scRNA and bulk sequencing data. Additionally, the association of the above genes with cellular senescence were also determined to support their heterogeneity in AD. A total of seven clusters were detected by known marker genes, including 5,990 excitatory neurons (*SNAP25*, *SYT1*, *SLC17A7*, *SATB2*), 5,874 inhibitory neurons (*SNAP15*, *SYT1*, *GADl*, *GAD2*), 4,794 astrocytes (*GFAP*, *AQP4*, *SLC1A2*), 4,198 microglial cells (*CSFlR*, *CD74*, *P2RY12*), 37,398 oligodendrocytes (*MOBP*, *MBP*, *MOG*), 2,731 oligodendrocyte progenitor cells (*PDGFRA*, *CSPG4*) and 487 pericytes/endothelial cells (*PDGFRB*, *CD248*). Astrocytes, microglia, and pericytes/endothelial cells emerged as the most active cell subtypes. DEG analysis showed similar results with those obtained in the scRNA sequencing analysis. The current study revealed the top 3 genes with increased expression levels in the seven cell subtypes. Further analysis showed that there was no change in the number of cells of a given cell type over total cells. The above finding indicated that the cell aging- and death-mediated decrease in cell count in AD was not reflected in the total proportion of cells. Therefore, additional data or replication studies are needed to verify the above results. Furthermore, the current study aimed to explore the dysregulation of cellular senescence based on single cell transcriptomic analysis. The results demonstrated that *RUNX1* was mainly expressed in microglia, *YAP1* in astrocytes and *NOTCH3* in pericytes/endothelial cells. The aforementioned active cell types were selected based on their activity scores.

In addition, herein, functional enrichment annotations of the shared active cell subtype-specific genes, bulk sequencing DEGs and cellular senescence-related genes were constructed. These three gene sets did not share any overlapping genes, thus indicating that the dysregulation of cellular senescence genes in AD could be complex and multifactorial, potentially involving multiple molecular pathways and cellular processes. The lack of overlapping parts also highlighted the significance of integrating multiple omics analysis to gain a comprehensive understanding of the molecular mechanisms underlying cellular senescence in AD. This study focused on the AD-associated cellular senescence genes via determining the interaction between cellular senescence genes and active cell subtype-specific genes or bulk sequencing DEGs. This approach could allow the identification of cellular senescence genes that could be specifically dysregulated in AD, thus providing a more targeted approach for the identification of potential therapeutic targets for AD.

The identified AD-associated cellular senescence genes were astrocytes, microglial cells and pericytes/endothelial cells, thus indicating that the dysregulation of the cellular senescence-related genes in the brain of patients with AD could involve multiple cell types. Astrocytes, microglial cells and pericytes/endothelial cells contained 37, 56 and 39 AD-associated cellular senescence genes, respectively. Based on analysing the human transcription factors from the HumanTFDB database, 11,12 and 10 AD-associated cellular senescence genes in the Astrocytes, Microglia and Pericytes/Endothelial subgroup were identified. Among these genes, *NR2E1, ID4, MXD4,* and PIVEP1 in astrocytes are believed to play roles in cellular transcription, differentiation, and regulation; however, their functions in AD-related cellular senescence remain unclear. In microglia, research on the roles of *PBRM1, RUNX1,* and *IRF5* in AD is limited, and to date, no studies have addressed their involvement in AD-associated cellular senescence. In pericytes and endothelial cells, *TBX2, ETS1, BHLHE40,* and *ID1* function as transcription factors that regulate cell proliferation and differentiation, yet their roles and underlying mechanisms in cellular senescence in AD require further in-depth investigation. A protein–protein interaction (PPI) network among transcription factors were constructed in our study. These findings suggest that senescence of different cell types in brain may contribute to the initiation and development of AD. Multiple genes may play critical roles in AD progression. Further investigation into the mechanistic contribution of senescence in specific brain cell types could provide a theoretical foundation for identifying AD biomarkers and therapeutic targets.

lncRNAs play an essential part in the aging process by controlling DNA expression at all levels. All non-coding RNAs are more specific for specific tissue types than protein-coding RNAs, highlighting their role in regulating tissue identity and function (Sherazi et al., 2023). Moreover, lncRNAs affect key cellular processes such as proliferation, differentiation, senescence and immune response, and play a significant role in several molecular processes associated with AD (Kim et al., 2017; Lettieri-Barbato et al., 2022). Furthermore, a regulatory network was constructed specifically aiming to elucidate the role of lncRNA-miRNA interactions in the regulation of AD-associated cellular senescence genes. The identification of lncRNA-miRNA interactions regulating AD-associated cellular senescence genes could provide a more comprehensive understanding of the molecular mechanisms underlying cellular senescence in AD and highlight the significance of non-coding RNAs in the pathogenesis of AD. Using the DGIdb database, we identified high-confidence cellular senescence genes in specific cell types: astrocytes (*GAPDH*, *GRK6*, *HMGB1*, *VEGFA*); microglia (*CDK1*, *CDKN1A*, *CSNK1A1*, *EWSR1*, *GRK6*, *PIM1*, *PRKCD*, *TOP1*); and pericytes/endothelial cells (*CDKN1A*, *CSNK1A1*, *GRK6*, *HSPA5*, *TOP1*, *VEGFA*), and a network diagram illustrating drug, gene, and miRNA interactions is presented. These findings provide potential targets for Alzheimer’s disease drug research.

## 5 Limitations

Based on the existing single-cell and transcriptome data, there is no genomic intersection has been found. Therefore, it could be necessary to redesign or employ new analytical methods (eg. WGCNA) for the DEGs or to investigate additional datasets and utilize new datasets, such as epigenomic or proteomic datasets, to further explore the results of this study. The AUCell database exerts several limitations, such as its dependence on the completeness of the senescence gene set used, thus potentially affecting the robustness of the findings, particularly in terms of cell population activity scores. Our study focused exclusively on the interactions among lncRNAs, miRNAs, and senescence genes. Extending the analysis to other types of ncRNAs, such as circular RNAs (circRNAs), would significantly enhance our understanding of the interactions between ncRNAs and senescence genes. It is necessary to focus on a specific cell type and perform further experimental validation of the identified key genes using cell lines or animal models. Pathway analysis in cell-based settings, active protein enrichment assays and *in vivo* or *in vitro* assessment of the candidate drugs could be beneficial to deeply clarify the molecular pathogenesis of cellular senescence and potential therapeutic drugs.

Overall, the present study integrated multiple omics analysis to highlight the significance of understanding the role of cellular senescence in the pathogenesis of AD. The development of novel therapies for AD is a complex and challenging task. However, the identification of potential therapeutic targets and drugs is a promising approach that could revolutionize the treatment of this devastating condition.

## 6 Conclusion

Overall, the present study integrated multiple omics analysis, 7 clusters by known marker genes and AD-associated cellular senescence genes in the Astrocytes subgroup (*SOX5*, *AR*, *HMGB1*, *NR2E1*, *ID4*, *TP53*, *MXD4*, *FOS*, *BHLHE40*, *PIVEP1*), microglia subgroup (*BCL6*, *ETS2*, *CEBPB*, *MXD4*, *FOS*, *NFE2L2*, *FOXO3*, *IRF3*, *PBRM1*, *RUNX1*, *IRF5*, *ZNF148*) and pericyte/endothelial cell subgroup (*SOX5*, *BCL6*, *ETS2*, *CEBPB*, *FOS*, *TP63*, *TBX2*, *ETS1*, *BHLHE40*, *ID1*) were identified. Our research has clarified the transcription factors and protein–protein interaction of AD-associated cellular senescence genes in the subgroups. The lncRNA-miRNA interactions in the regulation of AD-associated cellular senescence genes and the interaction between drugs and genes were constructed. These findings highlight the significance of understanding the role of cellular senescence in the pathogenesis of Alzheimer’s disease. The development of novel therapies for AD is a complex and challenging task. However, the identification of potential therapeutic targets and drugs is a promising development that could revolutionize the treatment of this devastating condition.

## Data Availability

The datasets presented in this study can be found in online repositories. The names of the repository/repositories and accession number(s) can be found in the article/Supplementary material.
