# Supplementary material for: Expression and potential regulatory mechanism of cellular senescence-related genes in Alzheimer’s disease based on single-cell and bulk RNA datasets
Source: Front Neurosci. 2025 May 21;19:1595847. doi: 10.3389/fnins.2025.1595847 (PMC12133858; doi:10.3389/fnins.2025.1595847)
Supplement: Supplementary file 1 [file Supplementary_file_1.zip › Supplementary Files/Supplementary Material.docx]

Supplementary Material

# Supplementary Data

Table S1. The basic information about human prefrontal cortex.

Table S2. Functional enrichment results of highly expressed genes specific to active cell subpopulations. (Supplementary material of Figure 2)

Table S3. DEGs of Bulk RNA-Seq data (AD vs Control). (Supplementary material of Figure 3)

Table S4. The enrichment function of scRNA seq active cell subsets intersects with the enrichment results of Bulk RNA Seq DEG. (Supplementary material of Figure 3)

Table S5 Gene-Drug interaction data in the DGIdb database. (Supplementary material of Figure S3)

Figure S1 The demographic data of the sample.

Figure S2. Dysregulation analysis in active cell subtypes. (A)Astrocytes. (B)Microglia. (C) Pericytes/Endothelial.

Figure S3. The interaction relationship among drugs, genes and miRNA. (A) Astrocytes. (B) Microglia. (C) Pericytes/Endothelial.
