# Supplementary figures and images for: Expression and potential regulatory mechanism of cellular senescence-related genes in Alzheimer’s disease based on single-cell and bulk RNA datasets
Source: Front Neurosci. 2025 May 21;19:1595847. doi: 10.3389/fnins.2025.1595847 (PMC12133858; doi:10.3389/fnins.2025.1595847)

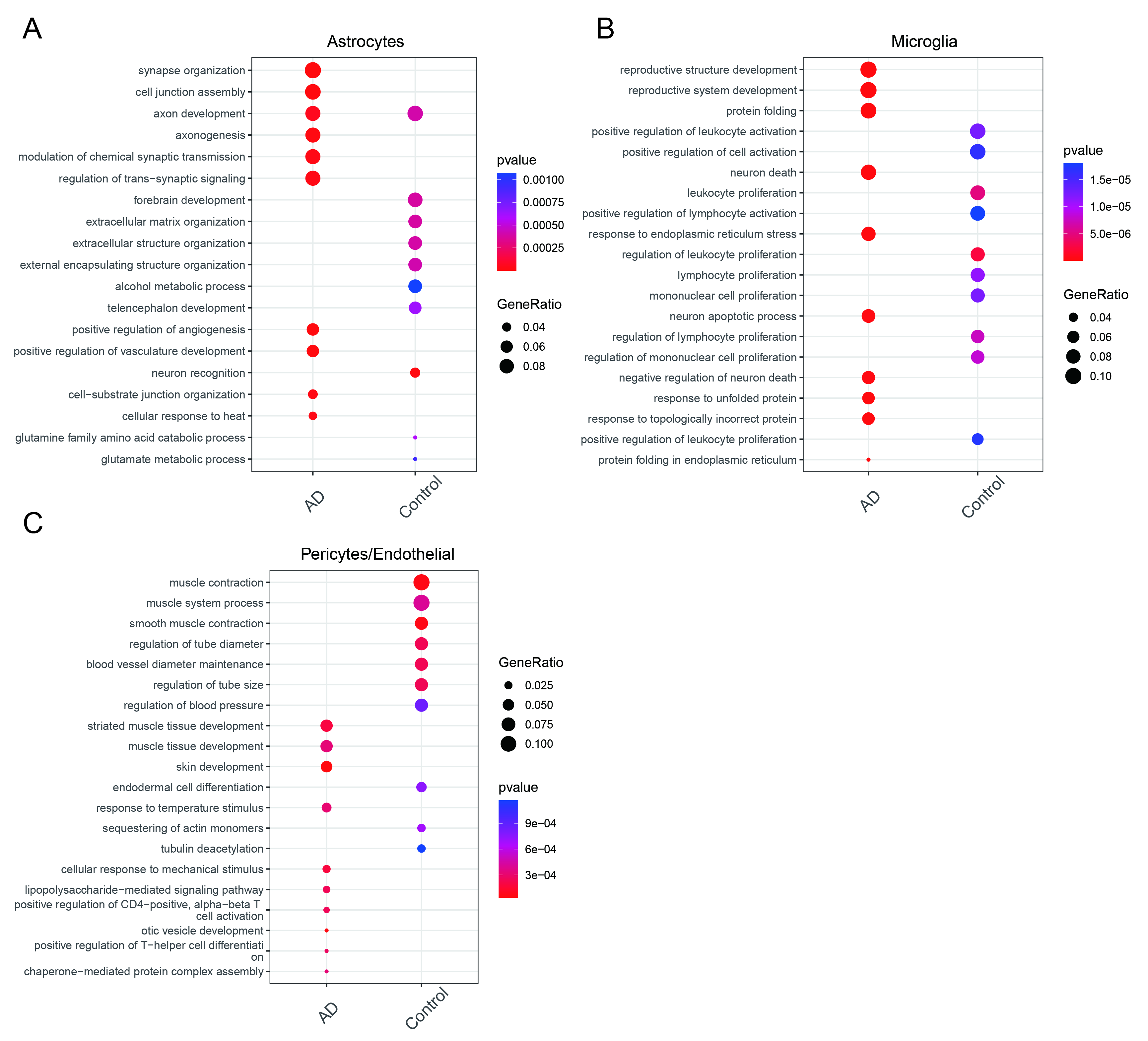

Supplement: Supplementary file 1 [file Supplementary_file_1.zip › Supplementary Files/Fig_S2.tif]

| Diagnosis | Sample size | Male (n) | Female (n) | Mean Age | Age SD |
|-----------|-------------|----------|------------|----------|--------|
| Control   | 48          | 28       | 20         | 80.90    | 8.42   |
| AD        | 47          | 22       | 25         | 84.51    | 6.51   |

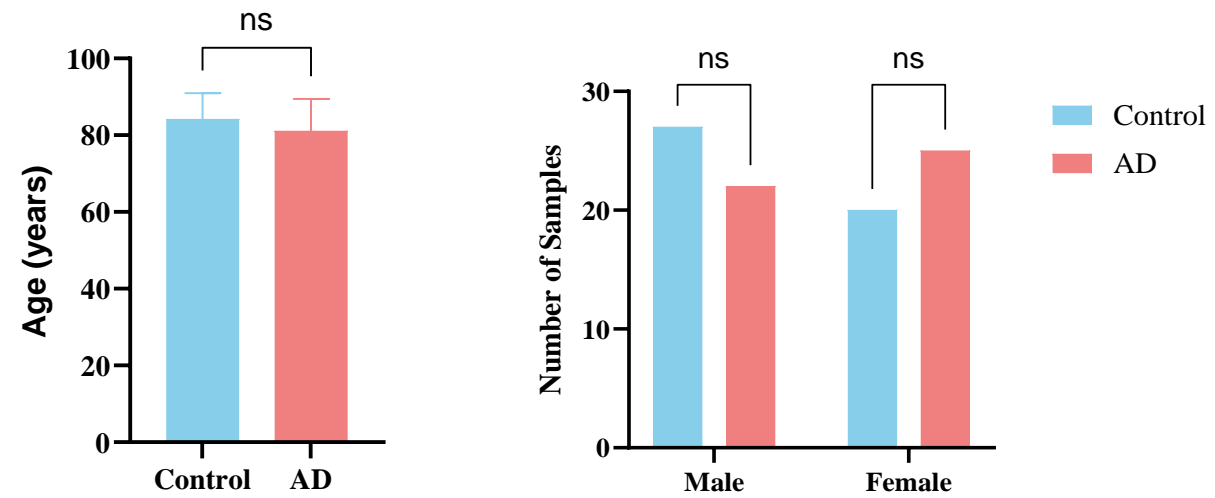

Supplement: Supplementary file 1 [file Supplementary_file_1.zip › Supplementary Files/Fig_S1 sample demographics.pdf]

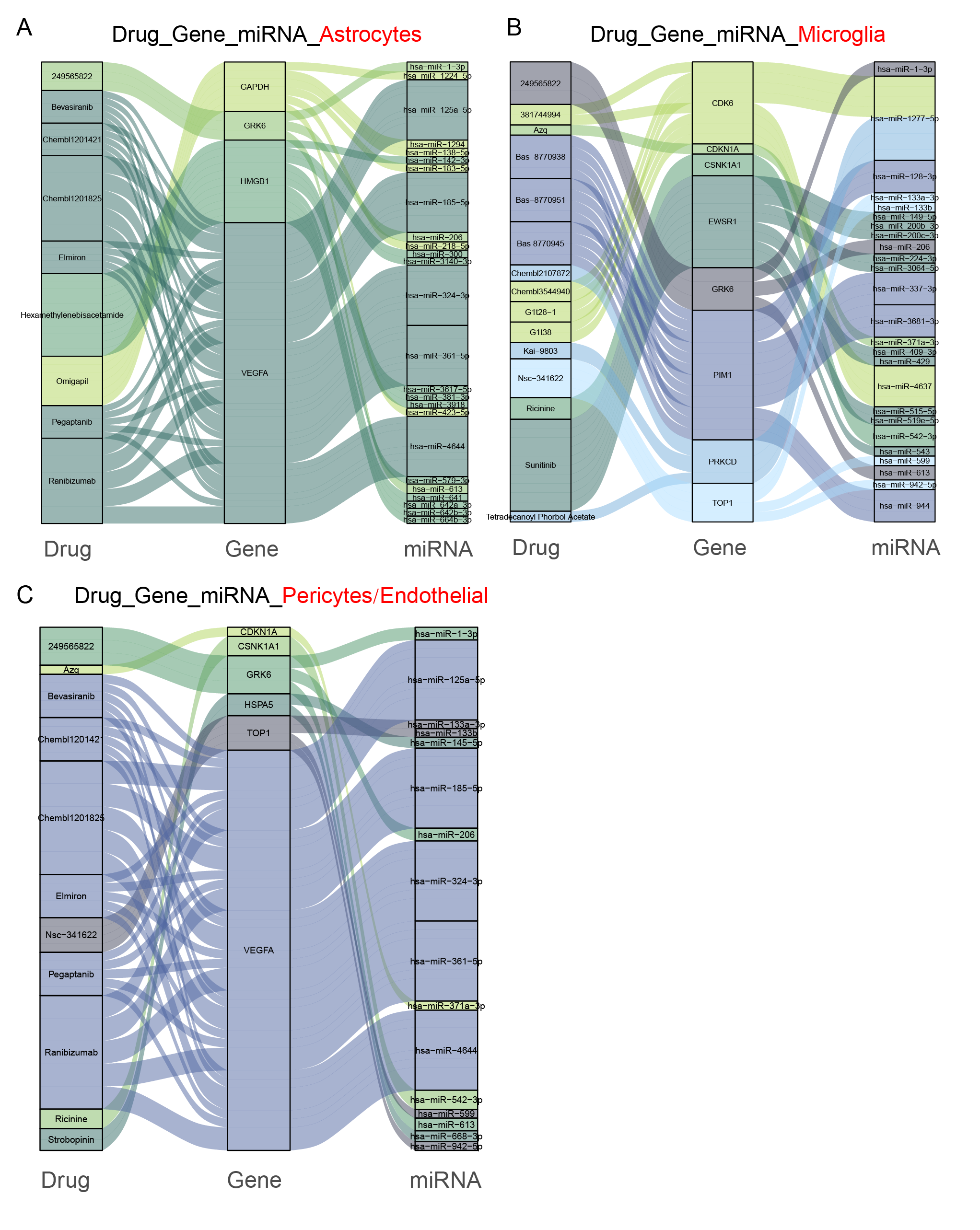

Supplement: Supplementary file 1 [file Supplementary_file_1.zip › Supplementary Files/Fig_S3.tif]
